# Is the market biased in M&A, dividend payment, and share repurchase events?

**DOI:** 10.1016/j.heliyon.2024.e29400

**Published:** 2024-04-12

**Authors:** Luu Thu Quang

**Affiliations:** Ho Chi Minh University of Banking, Ho Chi Minh City, Viet Nam

**Keywords:** Market reaction, Firm performance, M&A, Dividend payment policy, Share repurchase

## Abstract

Companies with excess capital can opt to: pay dividends to shareholders, buy back treasury shares for short-term shareholder benefits, or pursue M&A investments for long-term shareholder returns. Using difference in differences approach of event research methods combined with unique manually collected data sets, this paper investigates the market bias for three events: M&A, share repurchase, and dividend payment. The results show that information was leaked to the outside 1 day before it was officially announced at all events. When observing the company's performance 3 years after the event announcement, we also find that the market reaction is biased in M&A and stock dividend payment events, but accurate in the cash dividend payment and share repurchases. In addition, the market has the strongest and longest reaction to the news of the company buying back shares; has the weakest reaction to the stock dividend payment; has the shortest reaction to cash dividend payment; has a negative reaction to the acquisition company's stock, and has a positive reaction to the target company's stock. Our research has provided empirical evidence on the market response to published information, and supports CEOs make the most accurate choices when the company has an excess cash flow.

## 1 Introduction

When companies possess surplus capital, they have several alternatives at their disposal: firstly, they can distribute dividends to their shareholders; secondly, they can opt to repurchase treasury shares, thereby enhancing immediate benefits for shareholders; finally, they may choose to engage in mergers and acquisitions (M&A) as a means of maximizing long-term returns for shareholders. Firms in Vietnam now face numerous obstacles when repurchasing shares due to the Securities Law, which went into effect on January 1, 2006. In particular, a firm must cancel the number of repurchased shares and lower the charter capital when it buys back shares. This represents the biggest barrier for firms because charter capital continues to be a significant factor in the legal framework governing how firms must conduct their operations. For instance, the reduction of capital affects the capital adequacy ratio of the bank or affects the safe financial ratios of the insurance company, which must regularly report to the management agency. Or affect companies operating in industries that require minimum charter capital. The rule also stipulates that companies planning to repurchase treasury shares must have the general meeting of shareholders' approval, and companies are also prohibited from issuing shares for capital increases within six months after purchasing treasury shares. This prevents share repurchases from being made immediately when the company's shares are undervalued. It also limits the board of directors' flexibility in implementing the financial plans by issuing shares to raise capital. In general, the above regulations are not in line with international regulations because the majority of countries in the region, or developed countries, do not have mandatory regulations to reduce charter capital when buying back the shares.

A study by The Center for Capital Markets Competitiveness [1] which was conducted on 10,000 listed companies over 17 years in the US, shows that share buybacks serve many purposes than paying cash dividends. First, increasing stock market liquidity, especially in the case of a crisis or near-crisis. This becomes even more important in emerging or frontier markets when financial institutions play a small role, as they do in Vietnam. Second, reduce the volatility of stock prices. The company's flexibility in buying and selling treasury shares will lessen stock price volatility, particularly in the downward direction. Third, increasing the benefits for individual investors in the long term. Over time, more and more companies around the world began to prefer buying treasury shares over paying dividends in cash. Fourth, benefit all shareholders in times of crisis. Buying back shares of a company when the market price falls below its intrinsic value helps to increase the stock price. After that, the company can distribute the benefits by issuing repurchased shares to shareholders, or reselling them at a higher price once the market has stabilized, which will bring capital surpluses for the company and its shareholders.

Another option when the companies have excess capital is that they can perform M&A deals to increase future revenue and profits, thereby maximizing shareholder benefits instead of using that capital to pay dividends or repurchase treasury shares. In addition, M&A generates additional synergistic value by assisting companies in expanding into new markets, cutting labor costs, and utilize each other's technology to create competitive advantages.

The market will react to these events whether the company makes an M&A, pays a dividend, or buys back treasury shares. In the past, there have been many studies on market reaction or firm performance after M&A implementation [[2], [3], [4]]. [2] explore the contrasting responses of domestic and foreign institutional investors to M&A events in light of social responsibility. Others delve into market reactions related to information transparency in M&A deals. Additionally, some focus on the telecommunications sector, examining its dynamic features-such as regulatory shifts, rapid technological advancements, globalization, and fierce competition [5]. Other certain studies investigate market reactions to M&A activities influenced by CEO power [6]. Similarly, there are numerous studies on market reactions to dividend payout [7,8] or share repurchase events [[9], [10], [11]]. While [12] focuses on analyzing market reactions in two scenarios: increased dividend rates and decreased dividend rates, then [13,14] investigate the differences in market reactions in case of cash dividend and stock dividend. Another group of researchers explores the decision to pay dividends in relation to the growth potential of future company performance [15,16]. In the context of researching repurchasing treasury shares [17], aimed to understand the characteristics of companies that frequently engage in such repurchases. Meanwhile, another group of researchers investigated the motivations behind businesses repurchasing treasury shares [18], as well as the company's valuation during these repurchase transactions [17].

However, no research has been conducted to determine whether the market reaction to these events is biased or accurate, and no study has yet compared the intensity of the market response and the variations in firm performance across the three different types of events. Starting from the above research gap, in this work, the authors try to compare the difference in the magnitude of market response to various events and compare the operating performance after the company announces M&A, dividend payment, and share repurchase. This study contributes to the literature in the following ways: (1) this paper investigates the market bias for three events: M&A, share repurchase, and dividend payment. (2) This paper also compares the size of market reaction and operating performance of firms after implementing the above events. (3) The majority of earlier studies have been carried out in developed or emerging markets, where there are few constraints on the conditions of share repurchase or M&A. Since the legal framework for M&A activities has not been fully developed and strict requirements on share repurchase exist in Vietnam, where our study is conducted, it will add yet another angle to the discussion of this issue. (4) Extending on the previous studies, our study divides dividends into 2 cases: cash dividends and stock dividends. M&A is also divided into 2 cases: the case of the acquiring company and the case of the target company. (5) Finally, this study exploits a unique set of manually collected data for M&A events, share repurchases, and dividend payments based on information published by the mass media.

By applying the event study research method combined with the cumulative abnormal return (CAR) to assess market reaction, we provide evidence that the market reacts positively to share buyback information, cash dividends, and stock dividends. For M&A information, the market responds favorably to the shares of the target companies in M&A announcements, but negatively to the acquiring company. The results also indicate that the market has the strongest and longest reaction to the news of the company buying back shares; has the weakest reaction to the stock dividend payment; has the shortest reaction to cash dividend payment. The performance of the target company and the acquiring company has significantly changed before and after the M&A. There is proof that share repurchases and cash dividend payments are signs that firms will perform much better in the future. When observing the company's performance 3 years after the event announcement, we also find that the market reaction is biased in M&A and stock dividend payment events, but correct in the cash dividend payment and share repurchases.

There are five sections in this study. The first section provides a summary, background information, and an explanation of the topic selection. The paper reviews the theoretical basis and previous related studies in the second section. Section 3 presents data and research techniques. Section 4 presents the study findings and discussion. Finally, paper discusses the policy implications, limitations of the study and conclusion.

## 2 Literature review

Public companies listed on stock exchanges frequently engage in M&A, dividend payments, and share repurchases. As a result, many studies of these events have been conducted in the past. The author separates the literature into three subsections that correspond to three distinct events.

### 2.1 Previous studies on market reaction and corporate performance to M&A events

Numerous studies have evaluated how the market responds to M&A announcements. However, the outcomes are very different when looking into each study. For example [3], investigated the short-term reaction of the market and the role of institutional investors to M&A deals in the United Kingdom. The findings revealed that both domestic and foreign institutional investors have a negative reaction to M&A announcements [2]. study the market reaction to M&A activities under the impact of social responsibility, the results show that M&A deals done by companies with high social responsibility will receive positive reaction, while deals made by companies with weak social responsibility will receive a negative reaction. Agreeing with the above view [4], evaluate the transparency of information disclosure for M&A deals. The results show that stock prices respond positively to companies with transparent disclosure policies when implementing M&A [19]. claim that the stock market's response to an M&A event can be unfavorable if CEOs encounter some issues in the negotiations, such as agreements that generate over-payments for the M&A, or conflict of terms post-M&A. Meanwhile [5], examine M&A in the telecommunications sector. They argue that M&A in general does not benefit stock market participants mainly due to the dynamic characteristics in the telecommunications industry such as regulatory changes, rapidly evolving technology, globalization, and intense competition. Similarly [20], provide evidence that shareholders of acquired companies suffered a wealth loss of nearly 20 % in the post-merger period 1990–1994 [6]. investigated the relationship between CEO power, respective acquisition activities, and market reaction to M&A announcements. They conclude that when CEOs are in positions of great power, the market reacts negatively because these CEOs exhibit overconfidence, making it easy to implement M&A deals incorrectly. By using cumulative abnormal return (CAR) to gauge market reaction in a rumors-heavy environment [21], provide evidence that the market reacts positively to real M&A news but reacts negatively to inaccurate rumors about M&A. In addition, a variety of other studies have suggested that the market reacts positively to the acquired company but negatively to the acquiring company, and almost all profits from operations mergers are accrued to the shareholders of the target company while the acquirer's shareholders receive zero or negative returns [[22], [23], [24]].

The primary objective of mergers and acquisitions is to develop synergistic value and competitive advantage to boost future revenue and profits for both the acquirers and the target companies. Numerous studies explain why and how much M&A impacts corporate performance. For instance Ref. [25], contend that companies in the modern industrial era are primarily acquiring other companies based on technology and new digital innovation. In recent years, the main objective of M&A has been to create digital innovation efforts. Digital enhances business performance in the industrial age and helps companies gain a competitive advantage. From a cost perspective [26], believes that the value created through acquisitions will have a positive effect on the overall efficiency of the firm. Since implementation increases production costs explicitly included in the consolidated financial statements, this improvement will then be reflected in stock price movements. According to Ref. [27], a company's growth potential, proprietary assets, firm size, and company age all have a favorable effect on the performance of the company following mergers and acquisitions. However, after mergers and acquisitions, corporate governance, ownership, and solvency have no bearing on a company's performance. Meanwhile [28], did not find any specific relationship between company performance and M&A activities, but suggested that M&A effectiveness is a multifactorial structure. There is no one specific factor that captures all, the effectiveness of M&A is the link of the integrated process with the long-term performance of the company (including accounting and financial profitability) through customer retention and realizing synergy.

In contrast to the aforementioned findings [29], noted that when M&A is carried out by family-owned enterprises and affiliated company groups (cross-ownership), it will eventually result in a decline in the company's performance. In other words, the negative effects of the conservative mentality of ownership managers outweigh the benefit of reducing agency problems. Similarly [30], reported reduced profitability of companies due to M&A [31]. found a negative change in productivity, a significant downward trend in profits, a significant negative impact on revenue growth, and a shrinking workforce after the merger. Overall, they conclude that mergers harm corporate performance [32]. confirmed the negative impact of mergers on corporate performance. In particular, the values of Return on Assets, Return on Equity, and Return on Sales were significantly lower than they were before the M&A [33]. again examines the impact of executive compensation on company performance after mergers and acquisitions. Using financial and accounting performance measures, they show that CEO compensation generated by M&A negatively affects the performance of listed companies [34]. provide different evidence of performance for cross-border M&A and domestic M&A. In detail, domestic M&A performs better and cross-border M&A does not improve corporate financial performance. In order to explain this, they contend that performance after M&A is strongly correlated with exchange rates, infrastructure, labor costs, institutional gaps, and technology levels in the countries of the acquired company.

### 2.2 Previous studies on market reaction and corporate performance to share repurchase

Repurchasing shares is driven by a variety of factors, including the following: (1) It might occur when the CEO of the feels that the firm is being undervalued. (2) Or the company is trying to fend off hostile takeovers. (3) Or the company finds that the stock price has fallen too sharply, the repurchase of treasury shares can stabilize the market, thereby protecting investors. (4) Or just to decorate financial ratios [10,18,35]. [18] find that the market reacts more favorably to the share repurchase of undervalued companies [9]. investigates the market response following the company's actual share buyback on the Hong Kong market. He discovered that the market reacted positively to actual buybacks, with the 3-day CAR (cumulative abnormal return) being significant at 0.43 %. He also discovered that the market responded positively to actual share repurchases of small companies with high market book values. Supporting this view [17], agree that the market only reacts positively to the news of share repurchase of small companies with high BM ratios, while for big companies with low BM ratios, the stock price did not have any reaction [36]. are interested in the relationship between the company's reputation and the CEO's reputation on the stock market's reaction when the company announces the repurchase of treasury shares. In particular, when a company with a solid reputation announces a share repurchase on the market, the market will respond favorably. Companies with unreliable management, however, did not experience any market reaction when they disclosed information about their share purchase. According to Ref. [17], who are interested in both market reactions as measured by long-term abnormal returns and frequency (the number of times to repurchase treasury shares), the results show that the market reacts to companies more forcefully the more frequently share repurchases are made.

There is evidence that signaling theory, which discusses communication between firms and stockholders, can reduce information asymmetry [37]. When a company buys back treasury shares, it is a reliable signal that business is getting better [38]. [39] contend that a share repurchase announcement is a signal to the market from a company's executives to convey positive expectations about the company's performance going forward. This behavior is particularly noticeable in young businesses with excellent investment prospects. Similarly [40], use ROA, and ROE ratios to study the change in corporate performance around the share repurchases. The findings revealed that performance increased following share buybacks, and share buybacks are a sign of promising futures for young and growing businesses [11]. analyze the impact of extraordinary capital investment and financial flexibility on firm performance after share buybacks. The results indicate that firms with sufficient financial flexibility perform better than firms with insufficient financial flexibility after share buybacks. In other words, financial flexibility can reduce the investment crowding out effect and positively affect the performance of companies after share repurchase.

Contrary to the results of the above studies, the free cash flow theory suggests that the negative reaction of the market to the announcement may be connected with a decline in the firm's free cash flow. When a company buys back shares, it lowers its free cash flow and leaves less money available for investments in worthwhile projects that will boost the firm's productivity in the long run [41]. found little evidence of any improvement in operating performance following the share repurchase announcement. Similarly [42], based on a sample of more than 4000 share repurchase announcements, indicated a slight improvement in the announcement year's performance and found no evidence of an improved business performance in the following years. Meanwhile [43], analyzed the performance after share buyback in the context of the company's financial difficulties. Their results show that the company's abnormal profits and operating performance are significantly worse after performing a share buyback than companies without financial problems [44]. are interested in the CEO's call options affecting the decision to buy back treasury shares and the company's performance in the following years. The results show that companies in the group that offer a lot of call options to CEOs are often highly motivated to buy back treasury shares and often experience a decline in performance after the buyback. This implies that CEOs' repurchase decisions are significantly influenced by stock price concerns and that they may not act in the best interests of shareholders over the long term.

### 2.3 Previous studies on market reaction and corporate performance to dividend policies

When an efficient company generates a capital surplus, the company can choose to pay dividends to investors instead of buying back treasury shares. Dividends are divided into two types: cash dividends and stock dividends. Dividends in cash or shares continue to be divided into 2 cases: higher dividends and lower dividends compared to the previous year. Studies that support the dividend signaling theory suggest that the market usually responds positively to the higher dividend events and negatively to the lower dividend events. For example [45], argues that dividend increase announcement leads to an increase in stock returns of stock-traded companies, whereas the dividend reduction is associated with a decrease in stock returns [46].'s research also demonstrates that raising a company's dividend results in a favorable market response; conversely, cutting a dividend had little impact on the market [12]. divide cash dividends into two categories: higher dividends and lower dividends compared to the previous year. The results show that the negative reaction of the market to the announcement of dividend reduction is clearer than the positive reaction to the announcement of an increased dividend. According to Ref. [47], when underperforming firms disclose dividend information, the market reacts more favorably than when efficient firms announce the dividend payment. Meanwhile [13], are interested in stock dividends, and their findings indicate that the announcement of a stock dividend significantly increases the share price. The market's expectation that the company's future cash flow will increase right away is what caused the share price to increase after the announcement of a stock dividend. Similarly [48], also found the excess returns in ex-dividend date at the firms that issue new shares to pay dividends [49]. also find evidence that the market appears to respond favorably to the announcement of a stock dividend, and this reaction is stronger for a company that pays a high-proportion stock dividend at the same time as cash dividend information.

In contrast, some studies reject the dividend signal theory. For example [50], demonstrates that during the years from 2008 to 2010, the Russian market had a negative reaction to dividend increases of the companies with high growth prospects. Or very little evidence has been found to support the dividend signal theory in developed stock markets during the financial crisis [51,52]. [53] also discovered no evidence supporting the claims that companies that distribute stock dividends have better investment opportunities or that stock dividend announcements cause the market to decline. Using the event study method [54], observed stock prices 7 days before and 7 days after the company announced its stock dividend payment. The results indicated no appreciable positive abnormal returns on the stock dividend announcement occurred during the event period; there is no greater average abnormal return in the initial stock dividend issuers than in the subsequent stock dividend issuers; there is no greater average abnormal return in high stock dividend issuers than in low stock dividend issuers.

Dividend signal theory states that when a company increases its dividend payout ratio, it will signal improved corporate performance in the future. A lot of previous research has been implemented to investigate this theory. For example [55], examine the effects of ownership structure and dividend payout on firm profitability, valuation, and specific risk. They found that firms with highly concentrated ownership tend to have low dividend policies, which in turn leads to lower valuations and higher specific risk. The results also confirm that firms that pay regular dividends are generally less risky and therefore require high valuations [56]. use ROE to measure corporate performance, they also find evidence that when a company pays high dividends, it signals an increase in future ROE. Similarly [15,16], argue that paying a large amount of cash dividends will reduce agency problem and can improve firm performance [57]. also support the evidence that dividend policy has a significant positive effect on shareholder wealth and firm performance. In summary, many studies suggest that an increase in the cash dividend payout ratio will improve the stock price because they provide information about the future profitability of the company.

On the contrary, many studies do not support above view. Specifically [14], believes that paying cash dividends can reduce firm performance since a high cash payout policy can be driven by a scarcity of potential investment opportunities in the future. If a stock dividend is paid instead of cash dividend, the company may raise retained earnings, which unintentionally results in excess liquidity and creates a conflict of interest between shareholders and managers. Specifically, managers have the motivation to work on ineffective projects that may increase their benefit but lower the company's value [58]. [59] suggest that managers who are only concerned with their self-interest may be tempted to expand the size of the firm beyond the optimal level. If a company generates excess cash flow, managers are more likely to invest in inefficient projects. And the larger the free cash flow, the more likely agency costs will be incurred, resulting in a decrease in company efficiency. In line with the aforementioned viewpoint [60], found a negative correlation between dividend yield and shareholder wealth. The findings also revealed that leverage and dividend payout ratio have a negative impact on ROA. Meanwhile [61], argue that dividend payments may influence a company's performance positively or negatively. Therefore, the impact of the dividend payout ratio on corporate performance is still unclear.

[62] proposed a theory of behavioral bias in security markets, suggesting that irrational beliefs can significantly impact stock trading decisions without our conscious awareness. One common behavioral tendency is overconfidence. Traders afflicted by overconfidence exhibit excessive certainty in their abilities, leading them to make specific, misguided choices. For instance, they might purchase stocks based on optimistic growth potential, only to find that actual results diverge from their forecasts. Conversely, they may sell stocks they believe will perform poorly in the future, but when companies release information that contradicts their trading assumptions. In contrast to this biased reaction, it also exists accurate reactions in the market. These accurate reactions occur when the market trades in alignment with announced future business results. For example, the market experiences a price increase that is followed by a strong corporate earnings report [63,64].

By combining previous studies on market reaction and firm performance around announced events, we propose the specific hypothesis.

H1 The market reaction is considered bias (accurate) if it responds negatively (positively) to the events, which is accompanied by an improvement corporate performance several years after the event.

H2 The market reaction is considered bias (accurate) if it responds positively (negatively) to the events, which is typically accompanied by a decline in corporate performance several years after the event.

## 3 Data and methodology

The highlight of this study is that we use a unique set of hand-collected data on the announcement dates of share repurchase, M&A, and dividend payments on Vietnamese stock market. Data on the event announcement dates are collected from official information published in the mass media from 2006 (since the securities law takes effect) to 2022. In case the disclosure date is a weekend, the date used for analysis is the next working day. The data collection method includes the following steps: Step 1: To find information, we conduct a Google search for all relevant keywords, such as M&A, share repurchase, and dividend payment. Step 2 involves removing events of the companies that are not listed on the Vietnam Stock Exchange. Step 3, in order to confirm that the announcement date is the earliest date across all mass medias, we conduct a more thorough search for each event. Step 4, to ensure the accuracy of the information, we double check the information published on the website of the stock exchange. After being sure about the first announcement date, the final step is to import the stock ticker and announcement date into the general data set. The limitation of this method is that we might not be able to search all the information and therefore may miss some events. Datastream is used to gather additional information like total assets, leverage, book value, and the closing stock price after dividend adjustment. After removing observations with missing data, the total sample contains 217 companies that carried out a total of 657 events, including 296 dividend events (184 cash dividend events, 112 stock dividend events), 113 share repurchase events, and 248 M&A events (111 acquiring company events, 137 target company events).

This study uses the event study methodology to track how the market responds to announcement dates. The event study method is frequently employed to assess the effect of a financial or economic event on the stock price, profitability, or financial ratios of a company [65]. The ROA variable is used to gauge a company's performance. Abnormal return (AR) and cumulative abnormal return (CAR) are used as representative variables of market reaction.

To calculate AR and CAR, we implement the following steps sequentially. First, we need to calculate the actual return of all stocks announcing the event using the following formula (Eq (1)):

(Eq 1) $$R_{j,t}=\ln\left(\frac{P_{j}}{P_{j,t-1}}\right)\times 100$$

Where R_j,t_ is the return of stock j on day t; P_j,t_ is the closing price of stock j on day t. Next, we calculate the expected return of the companies based on the market model, by running a regression of stock returns against market returns (using VN Index) to control for overall market impact (Eq (2)). Based on earlier research [66], this study uses a window size of 252 days (window(-254, −2)) before the event because that is the typical number of trading days in a calendar year. As a result, this estimated time frame is appropriate because it includes all potential seasonal cycles throughout the year.

(Eq 2) $$R_{j,t}=\alpha_{j}+\beta_{j}R_{m,t}+\varepsilon_{j,t}$$

Where, R_m,t_ is the market return on day t as measured by VN Index. Next, we use the parameters α_j_ and β_j_ found from the formula (Eq 2) to calculate the expected return (ER) by least squares regression (Eq (3)) over the event time period (window(-1,+1), where day 0 is the day of the event announcement, day −1 is the day before the event 1 day, day +1 is the day after the event 1 day. Although there is no standardized length for an event period, but the event duration of −1 to +1 day is the most commonly used event duration to capture the response of the market for event studies [67].

(Eq 3) $${ER}_{j,t}={\hat{\alpha }}_{j}+{\hat{\beta }}_{j}R_{m,t}$$

Finally, using the equation in Eq. (4), the abnormal return (AR) is determined by deducting the expected return from the actual return during the event window (window (−1,+1)).

(Eq 4) $${AR}_{j,t}=R_{j,t}-{ER}_{j,t}$$

It is necessary to run more than one day of tests to get a complete picture of how events affect the market reaction. As a result, we also examine the cumulative abnormal return (CAR), which is calculated by aggregating all the abnormal returns of 3 days around the event (window (−1,+1) using the formula Eq 5. CAR is also used as the dependent variable in the regression analysis in the following sections.

(Eq 5) $${CAR}_{j,t}=\sum_{t=t-1}^{t1}{AR}_{j,t}$$

The primary purpose of the paper is to compare firm performance and market response to three different types of events. We create two a new variable with the name Different Indicator (DI), which is calculated by subtracting the CARs (or ROA) of the two events to be compared. DI is a shortened form of the Difference in Differences method. The advantage of this approach is that it removes biases in post-intervention period comparisons between the treatment and control groups. These biases could result from permanent differences between the groups, as well as biases from comparisons over time in the treatment group, which could be the result of trends due to other causes of the outcome [68]. DI is measured by Eq (6) or Eq (7):

(Eq 6) $${DI}_{CAR}={CAR}_{e1,w}-{CAR}_{e2,w}$$

(Eq 7) $${DI}_{ROA}={ROA}_{e1,w}-{ROA}_{e2,w}$$

In which, DI_CAR_ is the difference between of CAR of event e1 and CAR of event e2 in the window w. If DI is positive, it means that magnitude of market reaction to event e1 is stronger than event e2. Similarly, DI_ROA_ is the difference between of cumulative ROA of event e1 and cumulative ROA of event e2 in the window w.

Another purpose of this study is to examine the impact of different factors on the market reaction when companies disclose information about M&A events, dividend payments, and share repurchases. To achieve this goal, the variable CAR is used as the dependent variable to measure the 3-day cumulative abnormal returns around the event. The main independent variables include the size of the repurchase, dividends, and M&A. The proposed estimation model is as follows:

$${\;\text{Model}\;1:CAR}_{i,t}=\beta_{0}+\beta_{1}{CDIV}_{i,t}+\beta_{2}{ROA}_{i,t}+\beta_{3}{BM}_{i,t}+\beta_{4}{GO}_{i,t}+\beta_{5}{SIZE}_{i,t}+{\beta_{6}{LEV}_{i,t}+\varepsilon }_{i,t}$$

$${\;\text{Model}\;2:CAR}_{i,t}=\beta_{0}+\beta_{1}{SDIV}_{i,t}+\beta_{2}{ROA}_{i,t}+\beta_{3}{BM}_{i,t}+\beta_{4}{GO}_{i,t}+\beta_{5}{SIZE}_{i,t}+{\beta_{6}{LEV}_{i,t}+\varepsilon }_{i,t}$$

$${\;\text{Model}\;3:CAR}_{i,t}=\beta_{0}+\beta_{1}{M\&A}_{i,t}+\beta_{2}{ROA}_{i,t}+\beta_{3}{BM}_{i,t}+\beta_{4}{GO}_{i,t}+\beta_{5}{SIZE}_{i,t}+{\beta_{6}{LEV}_{i,t}+\varepsilon }_{i,t}$$

$${\;\text{Model}\;4:CAR}_{i,t}=\beta_{0}+\beta_{1}{REP}_{i,t}+\beta_{2}{ROA}_{i,t}+\beta_{3}{BM}_{i,t}+\beta_{4}{GO}_{i,t}+\beta_{5}{SIZE}_{i,t}+{\beta_{6}{LEV}_{i,t}+\varepsilon }_{i,t}$$

Where, CDIV is the cash dividend rate, is a dummy variable that takes the value of 1 when the company increases its cash dividend and the value of 0 when it decreases cash dividend. The SDIV, or stock dividend rate, is a dummy variable that takes the value of 1 when the company's stock dividend rate rises and the value of 0 when the stock dividend of the company falls. M&A is a merger and acquisition variable, a dummy variable, taking the value of 1 if it is the acquiring company, otherwise taking the value of 0 if it is the target company. REP is the degree of share repurchase, calculated by the total value of share repurchase divided by free cash flow. In addition, based on the previous theory, the additional control variables such as growth opportunity (GO) is proposed because [67] indicate that growth opportunities influence market response when the company announces the repurchase of treasury shares. We also add the financial leverage variable (LEV) to the model because previous literature has found that the leverage ratio significantly affects the market response to share buybacks or M&A executions. This is because investors perceive share buybacks or M&A by leveraged buyout strategy to pose a higher risk in the future [31]. MB ratio, firm size (SIZE), and ROA are additional variables that are included in the model because prior research has demonstrated their ability to explain changes in the market [69,70]. The Appendix contains specifics on how to calculate each variable.

## 4 Results and discussion

We first statistically describe all the variables in the model for different events. Table 1 divides events into five sub-categories including acquirers, target companies, share repurchase, cash dividend, and stock dividend companies. The average ROA in Panel A is 0.22, with a maximum value of 0.35, demonstrating that the companies make acquisitions often have high operating efficiency. Similar to this, the large firm size (mean of SIZE = 29.16), high valuation ratio (mean of MB = 2.31), and high financial leverage (mean of LEV = 0.36) demonstrate that companies engage in M&A that is high evaluated by market, sizable assets, but also extremely high leverage. The company might have financed M&A activities with loans. In contrast, the information in Panel B shows that the target companies are characterized by undervalued (mean BM = 0.71), poor performance (mean of ROA = 0.11), and small size (mean of SIZE = 24.64) and highly indebted (mean of LEV = 0.39). Panel C shows the mean of ROA = 0.25, which proves that companies implementing treasury stock repurchases have good performance. The mean MB = 0.51 proves that the CEO conducted share repurchase when they found their company undervalued, which is also consistent with the study of [36]. In addition, a remarkable feature of companies that carry out share buybacks is their mean LEV = 0.26, which is a relatively low debt ratio. It can be explained that because of lower borrowing rate, the company can reduce its annual financial operating expenses, thereby having enough capital to buy back shares when the company is undervalued by the market.

**Table 1:** Variable descriptions of the companies that conduct M&A, dividend payment, and share repurchase. ROA, MB, GO, SIZE, and LEV are calculated based on the most recent financial statements prior to the announcement of the event. Details on variable calculations are reported in the Appendix.

	CAR	ROA	MB	GO	SIZE	LEV
**Panel A: The acquiring firms**						
Mean	−3.42	0.22	2.31	1.25	29.16	0.36
Median	2.13	0.16	2.92	1.47	27.45	0.51
Max	14.22	0.35	4.15	2.13	29.96	0.62
Min	−15.29	0.12	1.17	0.54	25.50	0.28
St. deviation	5.21	0.17	2.25	1.14	28.47	0.55
**Panel B: The target firms**						
Mean	8.34	0.11	0.71	0.43	24.64	0.39
Median	12.90	0.09	0.65	0.32	24.09	0.40
Max	57.32	0.14	1.26	0.76	25.21	0.61
Min	3.55	−0.15	0.26	0.13	23.43	0.31
St. deviation	14.54	0.12	0.58	0.44	24.12	0.43
**Panel C: The share repurchase firms**						
Mean	9.84	0.25	0.51	0.38	25.62	0.26
Median	16.83	0.20	0.54	0.39	24.49	0.23
Max	23.59	0.41	1.43	0.96	26.36	0.36
Min	2.54	0.08	0.23	0.13	23.13	0.18
St. deviation	8.35	0.22	0.47	0.44	25.42	0.24
**Panel D: The cash dividend payment firms**						
Mean	0.34	0.28	2.26	1.17	26.73	0.34
Median	1.45	0.26	2.12	1.36	26.44	0.33
Max	2.84	0.39	3.79	2.19	27.16	0.40
Min	−1.48	0.18	1.27	0.42	23.77	0.13
St. deviation	0.89	0.24	2.33	1.15	26.42	0.31
**Panel E: The stock dividend payment firms**						
Mean	−0.83	0.15	1.51	0.59	26.82	0.35
Median	1.93	0.10	0.98	0.38	25.91	0.34
Max	4.21	0.21	2.97	0.96	27.44	0.48
Min	−5.59	−0.21	0.16	0.22	23.95	0.26
St. deviation	1.32	0.17	1.35	0.48	26.78	0.41

Table 1's panels D and E highlight the distinction between companies that distribute dividends in stock and cash. In particular, ROA demonstrates that a company that pays cash dividends performs much better than one that pays stock dividends (0.28 vs 0.15). Similarly, companies that pay cash dividends also receive high market attention compared to companies that pay stock dividends (MB = 2.26 vs 1.51). However, there is no significant difference in size and leverage ratio between these two companies.

The results from the above descriptive statistics can only provide the readers with an overview at the current time of the event, but they cannot provide information around the event, or later. For instance, how the performance or valuation of the company will change once it has acquired the other companies. In the next step, we will analyze in detail the market reaction and firm performance around the event announcement. Table 2 measures AR and CAR around the M&A announcement date. For the acquiring company, the results show that AR is statistically significant on days −1 (AR = −2.14 and p-value = 0.0371), day 0 (AR = −1.07 and p-value = 0.0194), date +1 (AR = −0.65 and p-value = 0.0011), day +2 (AR = −1.76 and p-value = 0.0569), day +3 (AR = −0.55 and p-value = 0.0782), date +4 (AR = −0.14 and p-value = 0.0237) and date +5 (AR = −1.21 and p-value = 0.0135). This implies that the market reacts negatively to an M&A event that begins on day −1 and lasts until day +5. It is possible to understand the negative market response that surfaced a day before the information was officially announced as a result of information leakage prior to the announcement. Similar to AR, when observing the time windows of CAR in Table 2, we see that window (−1,+1) and window (−1,+3) are statistically significant at the 1 % level. In addition, the window (−1,+5) is also statistically significant, which reinforces the evidence that the market's negative reaction lasts from 1 day before the announcement of the M&A to the 5th day after the date of the announcement.

**Table 2:** AR and CAR around M&A announcement. Day 0 is announcement day. *p < 0.1. **p < 0.05. ***p < 0.01

	Acquiring firms		Target firms	
	AR	p-value	AR	p-value
Day −5	0.26	0.1021	−1.09	0.1341
Day −4	−1.76	0.2813	−1.23	0.2123
Day −3	1.55	0.1983	−1.76	0.1746
Day −2	2.18	0.3390	−0.45	0.3294
Day −1	−2.14**	0.0371	2.08**	0.0174
Day 0	−1.07**	0.0194	1.2***	0.0024
Day +1	−0.65***	0.0011	1.77***	0.0016
Day +2	−1.76*	0.0569	2.65**	0.0451
Day +3	−0.55*	0.0782	1.93*	0.0652
Day +4	−0.14**	0.0237	0.67**	0.0347
Day +5	−1.21**	0.0135	−1.31**	0.0198
Day +6	0.58	0.2125	2.18	0.1245
Day +7	1.30	0.1980	0.99	0.1212
Day +8	−1.05	0.1783	−1.65	0.2213
Day +9	2.01	0.2090	2.01	0.2150
Day +10	0.08	0.3201	0.05	0.2891
Event window	CAR	p-value	CAR	p-value
Window (−5,-2)	2.23	0.1782	−4.53*	0.0821
Window (−1,+1)	−3.86***	0.0021	5.05***	0.0002
Window (−1,+3)	−6.17***	0.0009	9.63***	0.0042
Window (−1,+5)	−7.52*	0.0561	8.99**	0.0321
Window (−1,+7)	−5.64	0.2091	12.16	0.1021
Window (−1,+10)	−4.60	0.2283	12.57	0.2815

But when we observe the market's reaction to the target companies, the results are the opposite. Specifically, the market has reacted positively to the M&A news of the target companies from −1 to date +4, and the market starts to change on day +5. This result is reinforced by CAR as the window (−1,+1), window (−1,+3) and window (−1,+5) are all statistically significant. When the company releases information about M&A, the AR shifts from negative (−4.53) to positive (5.05). In conclusion, the AR and CAR statistical findings demonstrate that the market responds negatively to information about the acquiring company and positively to information about the acquired company.

The market reaction to the dividend announcement is then observed in Table 3. AR has a positive sign and statistical significance only on days −1, 0, and +1, demonstrating that the market has responded favorably to the news that the company will pay cash dividends. However, this reaction does not last long, only 3 days from day −1 to day +1. This argument is further supported by the statistical significance of the CAR result at the window (−1,+1) = 4.23. The reason for the favorable market response is that a company's decision to pay a cash dividend demonstrates that it is making real profits and having a positive cash flow. Similarly, the market also reacts positively to the news of paying stock dividends. In particular, AR has a positive sign and is statistically significant from −1 to +5. CAR results in the window (−1,+1), window (−1,+3), and (−1,+5) also confirm the positive trend of the market for stock dividend information. Additionally, we realize that the stock dividend payment has a longer positive period than the company's cash dividend payment. This response can be explained by the market's expectation that the company's free cash flow will increase immediately [13].

**Table 3:** AR and CAR around dividend payment announcement. Day 0 is announcement day. *p < 0.1. **p < 0.05. ***p < 0.01

	Cash dividend payment firms		Stock dividend payment firms	
	AR	p-value	AR	p-value
Day −5	0.17	0.2132	0.32	0.1532
Day −4	−0.77	0.2031	−1.76	0.1131
Day −3	1.28	0.1242	1.33	0.1082
Day −2	−2.55	0.3002	−2.38	0.3211
Day −1	0.98**	0.0231	1.18**	0.0200
Day 0	1.42***	0.0012	0.15***	0.0014
Day +1	1.83*	0.0561	1.55***	0.0064
Day +2	−1.65	0.2011	0.03**	0.0331
Day +3	1.70	0.1982	0.16**	0.0422
Day +4	1.03	0.3016	1.05**	0.0334
Day +5	−0.99	0.3546	−0.12*	0.0578
Day +6	2.10	0.1900	2.76	0.1443
Day +7	−1.66	0.2941	−3.16	0.2611
Day +8	2.19	0.3306	−1.67	0.3251
Day +9	0.08	0.1775	0.98	0.1500
Day +10	−1.25	0.1744	−1.42	0.1224
Event window	CAR	p-value	CAR	p-value
Window (−5,-2)	−1.87	0.1920	−2.49	0.1387
Window (−1,+1)	4.23***	0.0003	2.88***	0.0033
Window (−1,+3)	4.28	0.1222	3.07***	0.0012
Window (−1,+5)	4.32	0.2035	4.00**	0.0351
Window (−1,+7)	4.76	0.3712	3.60	0.3781
Window (−1,+10)	5.78	0.2277	1.46	0.2407

Table 4 presents the reactions of AR and CAR around the announcement of share repurchase. The results show that the market reacts very positively. Specifically, both window (−1,+1) = 6.45 and window (−1,+3) = 7.48 are significant at the 1 % level. In addition, window (−1,+5) and window (−1,+7) are also statistically significant, implying that the market reacted positively from day −1 until day +7. In general, this result supports previous studies, which suggested that the market will react more positively because investors believe that the share repurchase is a reliable signal that the company's performance will prosper in the future [9,17,36]. All the results in Table 2, Table 3, Table 4 show us that window (−1,+1) is the most statistically significant and also represents the most accurate market reaction, so the CAR variable in the interval window (−1,+1) will be used as the dependent variable in the regression model as commented by Ref. [67].

**Table 4:** AR and CAR around share repurchase announcement. Day 0 is announcement day. *p < 0.1. **p < 0.05. ***p < 0.01

	AR	p-value
Day −5	−2.10	0.1772
Day −4	1.66	0.3631
Day −3	−0.78	0.1359
Day −2	−1.62	0.2211
Day −1	2.05**	0.0313
Day 0	1.99***	0.0021
Day +1	2.41***	0.0032
Day +2	0.66**	0.0329
Day +3	0.37**	0.0463
Day +4	1.05**	0.0334
Day +5	1.11*	0.0577
Day +6	0.71*	0.0623
Day +7	−2.15*	0.0551
Day +8	−1.21	0.3244
Day +9	2.33	0.1230
Day +10	0.78	0.1227
Event window	CAR	p-value
Window (−5,-2)	−2.84	0.3212
Window (−1,+1)	6.45***	0.0019
Window (−1,+3)	7.48***	0.0040
Window (−1,+5)	9.64**	0.0214
Window (−1,+7)	8.20*	0.0562
Window (−1,+10)	10.10	0.1023

This study's main objective is to provide an answer to the following question: In order to get the best reaction from the market, should a company choose to pay dividends in cash, in stocks, buy back shares, or engage in M&A when it has excess cash? To answer this question, we need to compare how the market reacts to different events. For example, we calculated the difference indicator (DI) by subtracting the CAR of the share repurchase event from the CAR of the cash dividend payment event in the same time frame in order to compare the market reaction to both events. Do the same for other event comparisons. Panel A in Table 5 shows that the difference indicator (DI) is positive and statistically significant in both windows (−1,+1) and (−1,+3), implying that the market reacts more strongly to news of share repurchases than to news of cash dividend payments. Panel B also shows a positive and statistically significant DI, indicating that the market reaction is stronger when the company buys back shares than when the company issues shares to pay dividends. Similarly, Panel C shows us a greater magnitude of positive market response when a company pays cash dividends compared to stock dividends.

**Table 5:** Compare the market's responses between various events. The Difference Indicator (DI_CAR_) is calculated by subtracting the CARs of the two compared events.

	Difference Indicator	p-value
Panel A. Share repurchase vs Cash dividend payment		
Day (−1,+1)	2.22***	0.0042
Day (−1,+3)	3.2*	0.0711
Day (−1,+5)	5.32	0.1002
Day (−1,+7)	3.44	0.1004
Panel B. Share repurchase vs Stock dividend payment		
Day (−1,+1)	3.57***	0.0030
Day (−1,+3)	4.41*	0.0692
Day (−1,+5)	5.64	0.2011
Day (−1,+7)	4.6	0.2035
Panel C. Cash dividend payment vs Stock dividend payment		
Day (−1,+1)	1.35**	0.0021
Day (−1,+3)	1.21	0.1599
Day (−1,+5)	0.32	0.1077
Day (−1,+7)	1.16	0.2018

We next investigate whether the market reaction is biased against the company's future performance by looking at the average ROA and annual ROA over the three-year period before and after the event announcement. Table 6 shows that the ROA of acquiring companies tends to increase gradually from year −2 to year +3, implying that acquiring companies have increased operational efficiency after M&A implementation. In contrast, ROA in target companies tended to decrease gradually from 0.24 in the year −2 to 0.11 in the year +3. The average ROA of the period (−3, −1) = 0.21 is larger than that of the period (+1,+3) = 0.17, supporting the argument that the target company's performance is worsened after M&A. This result clearly shows that the market reaction has been biased against firm performance. In particular, the market reacts negatively to the acquiring company, but after the M&A, all of the acquiring companies increase their operating performance, whereas the target companies experience reduced performance, but the market responds favorably to them.

**Table 6:** Firm performance around M&A announcement. Year 0 is announcement year. ROA is calculated for 3 years before and after the event. *p < 0.1. **p < 0.05. ***p < 0.01

	Acquiring firms		Target firms	
	ROA	p-value	ROA	p-value
Year −3	0.17	0.1102	0.18	0.2016
Year −2	0.19*	0.0690	0.24**	0.0337
Year −1	0.22**	0.0215	0.20**	0.0410
Year 0	0.21**	0.0301	0.12*	0.0799
Year +1	0.23*	0.0566	0.19***	0.0071
Year +2	0.26**	0.0119	0.11*	0.0882
Year +3	0.24*	0.0561	0.11*	0.0656
Event window	Average ROA	p-value	Average ROA	p-value
Year (−3,-1)	0.19**	0.0121	0.21**	0.0128
Year (+1,+3)	0.24*	0.0677	0.17***	0.0007

Table 7 provides firm performance data around the dividend event. ROAs are significantly from year −1 to year +3 combined with average ROA in the period (−3, −1) = 0.18 is smaller than the window (+1,+3) = 0.26, showing the firms' performance has improved significantly since the announcement of cash dividend payment. In other words, the market reacted correctly to the dividend signal theory in the case of the cash dividend payment firms. But when a company paid the stock dividend, the market reaction is biased because there was no proof that the firms' performances changed before and after the stock dividend announcement (Both the average ROA for the period (−3,-1) and (+1,+3) are not statistically significant).

**Table 7:** Firm performance around dividend announcement. Year 0 is announcement year. ROA is calculated for 3 years before and after the event. *p < 0.1. **p < 0.05. ***p < 0.01

	Cash dividend payment firms		Stock dividend payment firms	
	ROA	p-value	ROA	p-value
Year −3	0.20	0.2123	0.19	0.2190
Year −2	0.18	0.1771	0.20*	0.0553
Year −1	0.15*	0.0772	0.17	0.3051
Year 0	0.24*	0.0866	0.17**	0.0210
Year +1	0.26**	0.0351	0.20	0.1677
Year +2	0.25**	0.0322	0.19	0.1015
Year +3	0.27*	0.0519	0.15	0.1909
Event window	Average ROA	p-value	Average ROA	p-value
Year (−3,-1)	0.18*	0.0562	0.19	0.1981
Year (+1,+3)	0.26**	0.0501	0.18	0.2017

Data on a firm performance around the share repurchase is reported in Table 8. The results show that the ROAs are statistically significant in most of the years surrounding the event. In more detail, ROA at year 0 = 0.25 is statistically significant at a 5 % level combined with average ROA in period (−3,-1) = 0.20 smaller than period (+1,+3) = 0.28, indicating that the company's operating efficiency improved in the year the company announced the share repurchase, and this performance continued to improve 3 years later. This result supports previous studies and shows that the market has correctly forecast corporate profits when it believes that the repurchase of treasury shares is a signal to improve the company's performance in the future. Table 9 presents the difference between firm performances cross various events. The companies that buy back shares have better performance than companies that pay cash dividends both pre and post event (Year (−3,-1) and Year (+1,+3) are positive in panel A). Similar results for share repurchase and stock dividend comparison pair (Panel B). Before the event announcement, companies that paid cash dividends have better performance than companies that paid stock dividends (Year (−3, −1) = −0.01 in Panel C). But there is a strong reversal after the event (Year (+1,+3) = 0.08). This demonstrates once more that issuing shares to pay dividends actually worsens agency issues rather than improving efficiency.

**Table 8:** Firm performance around share repurchase announcement. Year 0 is announcement year. ROA is calculated for 3 years before and after the event. *p < 0.1. **p < 0.05. ***p < 0.01

	ROA	p-value
Year −3	0.19**	0.0170
Year −2	0.21*	0.0536
Year −1	0.19*	0.0500
Year 0	0.25**	0.0293
Year +1	0.26**	0.0174
Year +2	0.29**	0.0152
Year +3	0.25*	0.0806
Event window	Average ROA	p-value
Year (−3,-1)	0.20**	0.0441
Year (+1,+3)	0.28**	0.0229

**Table 9:** Compare the firm performances between various events. The Difference Indicator (DI_ROA_) is calculated by subtracting the ROAs of the two compared events.

	Difference Indicator	p-value
Panel A. Share repurchase vs Cash dividend payment		
Year (−3,-1)	0.02**	0.0129
Year (+1,+3)	0.02***	0.0011
Panel B. Share repurchase vs Stock dividend payment		
Year (−3,-1)	0.01**	0.0130
Year (+1,+3)	0.10***	0.0071
Panel C. Cash dividend payment vs Stock dividend payment		
Year (−3,-1)	−0.01**	0.0230
Year (+1,+3)	0.08**	0.0179

Table 10 presents the regression results with the dependent variable CAR as explained earlier because it is an accurate proxy for market reaction to events such as M&A, dividend payment, and share repurchase. In model 1, the M&A variable is a dummy variable that shows the different degrees of impact of the acquiring company and the target company on the market reaction. The coefficient of M&A = −0.2461 is significant at the 5 % level (Model 1), indicating that the target companies' M&A information has a greater impact on market reaction than that of the acquiring companies. In other words, the impact of the target firms on market is 0.2461 greater than the acquiring company's.

**Table 10:** The results of the regression analysis. All data are normalized using the zero mean and unit variance method. CAR_i,t_ is dependent variable, is sum of abnormal return in event window (−1,+1). ROA, MB, GO, SIZE and LEV are measured on the last financial report prior to the announcement. The explanatory variables are listed in Appendix. T ratio is in parentheses. *, ***, *** at the 10 %, 5 % and 1 % significance levels, respectively.

CAR_i,t_	Model 1	Model 2	Model 3	Model 4
Constant	2.1092** (2.067)	1.9244* (1.992)	2.2076** (2.001)	1.7885*** (3.541)
M&A	−0.2461** (−2.112)			
CDIV		1.3083** (2.182)		
SDIV			0.8911** (2.0933)	
REP				0.6712*** (2.991)
ROA	0.2910* (1.883)	0.5204* (2.142)	0.3442 (1.245)	0.1559 (1.552)
MB	−0.4231** (−2.106)	−0.5791 (−0.889)	−0.3775* (−1.924)	−0.5562** (−2.133)
GO	−0.2763 (−1.227)	−0.3390 (−0.992)	−0.4003 (−1.346)	−0.1295 (−1.492)
SIZE	0.7322** (2.563)	0.6877* (2.141)	0.1427* (1.698)	0.6634** (2.455)
LEV	0.4586 (0.774)	0.3134 (0.8823)	0.6630* (1.779)	0.2784 (1.258)
Adjusted R^2^	0.2109	0.2676	0.1885	0.2125
Observation	248	184	112	113

Model 2 presents the impact of the cash dividend event on the market reaction. The coefficient of the CDIV dummy variable, which represents the difference in the market's response to an increase or decrease in the cash dividend ratio over the previous year, is 1.3083 (t ratio = 2.95). Since the market response is 1.9244 (the slope) for companies that reduce the dividend rate (the benchmark group), the size of the market response for firms that increase the cash dividend is 1.3083 + 1.9244 = 3.2327. This suggests that the market reaction is stronger for companies that increase cash dividends (3.2327) than for companies that reduce cash dividends (1.9244). This result is inconsistent to Ref. [12] when arguing that the negative market reaction to the announcement of a dividend reduction is more obvious than the positive reaction to the announcement of a dividend increase. Model 3 presents the effect of stock dividend payment on the market response. The fact that the company increased its dividend payout ratio per share has a greater impact on the market's response than the news that it decreased its stock dividend rate is indicated by the positive coefficient of the variable SDIV, which has a positive value and is statistically significant at 1 %. Model 4 measures the effect of stock buyback size on market response. The coefficient of the variable REP has a positive sign, showing that the larger the sizes of the share repurchases, the more positive impacts on the market's reaction. This can be explained in two ways: (1) when the company increases the scale of share repurchase, the demand for those shares raises quickly, which has a strong effect on the market. (2) When the company announces share repurchase, it will convey to the market the message that the company is undervalued, which will attract many investors. This argument is reinforced when looking at the MB coefficient (−0.5562) in model 4. In case the company buys back shares, the lower the company's valuation, the stronger the market reaction.

To mitigate the impact of exogenous factors, such as firm size, liquidity, macroeconomic trends, industry dynamics, and regulatory changes, on our analysis results, we employ the Difference-in-Differences (DID) method. This approach involves an additional comparison group, known as the control group. We created this control group to match the number of companies engaged in M&A, share repurchases, and dividend payments. Furthermore, we carefully selected control group companies with identical characteristics to those performing M&A, share repurchases, and dividends. Subsequently, we observed the market reaction for both the control group and the experimental group simultaneously. Our findings reveal a significant difference between the two groups, which is statistically significant at the 1 % level. Notably, the market exhibited no reaction during the same period for the control group. This proves that the market response observed in the experimental group is entirely influenced by M&A information, share repurchases, and dividend payments, without any impact from other extrapolated factors.

## 5 Conclusion

In contrast to other researches carried out in highly mature markets, this study is conducted in an environment where there are gaps in the legislative framework, restrictions on share purchases, M&A transactions, and the issuance of dividend-paying securities. This study assesses the market effect across three different types of events and examine business performance around the event announcement time by combining an event research technique with a distinctive collection of hand-collected data. Divided into two categories, acquirer and target firms for M&A transactions, and stock dividend and cash dividend firms for dividend payment transactions, respectively. The findings indicate that high operating efficiency, sizeable assets, and a high leverage ratio in the most recent financial statement period prior to the event announcement are frequent characteristics of the acquiring companies. This finding may be related to the fact that companies have used loans to finance M&A activities. On the other hand, the target firms stand out for their small size, poor performance, and undervaluation by the market. Meanwhile, companies that buy back shares typically have outstanding performance and lower debt ratios. CEOs buy back shares when they realize their company is undervalued. The finding also reports that the performance of cash dividend companies is significantly higher than that of stock dividend companies.

When observing the market reaction around the announcement date, we notice that information is leaks to the outside 1 day before it is officially announced at all events. This finding is consistent to Ref. [71]. The market reacts negatively to the news of M&A by acquiring companies starting from −1 and lasting until +5. In contrast, the market responds favorably to the target firms' M&A information. The news of a cash dividend, stock dividend, and share repurchase all causes positive market reactions, but the stock dividend payment's positive effects persist longer than the cash payment's positive effects.

The market reacts most positively and strongly in case the company announces to buy back treasury shares rather than cash dividends payment, shares dividend payment, or engaging in M&A. The regression model's variables M&A, CDIV, SDIV, and REP can all be used to explain the market response. In particular, the size of treasury stock repurchases positively impacts market response; the target company has a stronger impact on the market than the acquiring company; companies that increase the cash dividend have a stronger impact on the market than companies that reduce the cash dividend; and an increase in share dividend payout ratio has a stronger impact on the market than a company that decreases stock dividend ratio. All these finding are consistent to [5,6,12,18,20,35,47].

When observing the company's performance 3 years after the event announcement, we find that the market reaction is biased in M&A. The market reacts negatively to the acquiring company, but after the M&A, all of the acquiring companies increase their operating performance, whereas the target companies experience reduced performance, but the market responds favorably to them. Similarly, to this, when a corporation pays a stock dividend, the market's response is biased, but it is accurate when the company pays the cash dividend or buys back shares.

Research highlights the critical role of informed decision-making for CEOs dealing with surplus capital. Rather than conventional options like dividends or M&A, this study emphasizes share repurchases. By understanding market dynamics, CEOs can make well-informed choices, benefiting both the company and shareholders, this consist to Ref. [72] that companies pay close attention to various stakeholders, particularly external stakeholders, and wish to increase trust and transparency. Future research could explore long-term effects of share repurchases in different economic contexts.

## Funding statement

This study is supported by Ho Chi Minh University of Banking.

## Ethics and statement

Our study does not involve humans or animals.

## Data availability statement

Data will be made available on request.

## Additional information

No additional information is available for this paper.

## Consent for publication

Not applicable.

## Availability of data and materials

The datasets used for this study are not publicly available as they are collected at high cost but are available from the corresponding author on reasonable request.

## CRediT authorship contribution statement

**Luu Thu Quang:** Writing – review & editing, Writing – original draft, Supervision, Methodology, Investigation, Data curation, Conceptualization.

## Declaration of competing interest

The authors declare that they have no known competing financial interests or personal relationships that could have appeared to influence the work reported in this paper.

## Figures and Tables

**Table:** 


Variables	Abbreviation	Description
Abnormal return	AR	Calculate using Eq 4
Cumulative abnormal return	CAR	Sum of abnormal return in event window (−1,+1)
Cash dividend ratio	CDIV	A dummy variable, take the value 1 If the company increases its cash dividend and take the value 0 if it decreases its cash dividend.
Stock dividend ratio	SDIV	A dummy variable, take the value of 1 if the company increases its stock dividend, and take the value 0 if it decreases its stock dividend.
Mergers and acquisitions	M&A	A dummy variable, take the value of 1 if it is the acquiring company, and take the value of 0 if it is the target company.
Firm performance	ROA	Return on assets
Firm valuation ratio	MB	Market capitalization to book value
Share repurchase size	REP	Ratio of share repurchase to free cash flows
Growth opportunity	GO	Firm market value divided by book value of total assets
Size of firm	SIZE	Nature log of total assets
Leverage	LEV	The debt to total assets
